# Sepsis-associated encephalopathy: a vicious cycle of immunosuppression

**DOI:** 10.1186/s12974-020-1701-3

**Published:** 2020-01-10

**Authors:** Chao Ren, Ren-qi Yao, Hui Zhang, Yong-wen Feng, Yong-ming Yao

**Affiliations:** 1 Trauma Research Center, Fourth Medical Center of the Chinese PLA General Hospital, Beijing, 100048 People’s Republic of China; 2 Department of Burn Surgery, Changhai Hospital, The Navy Medical University, Shanghai, 200433 People’s Republic of China; 3 Department of Critical Care Medicine, The Second People’s Hospital of Shenzhen, Shenzhen, 518035 People’s Republic of China

**Keywords:** Sepsis-associated encephalopathy, Immune suppression, Vicious cycle, Therapeutic target

## Abstract

Sepsis-associated encephalopathy (SAE) is commonly complicated by septic conditions, and is responsible for increased mortality and poor outcomes in septic patients. Uncontrolled neuroinflammation and ischemic injury are major contributors to brain dysfunction, which arises from intractable immune malfunction and the collapse of neuroendocrine immune networks, such as the cholinergic anti-inflammatory pathway, hypothalamic-pituitary-adrenal axis, and sympathetic nervous system. Dysfunction in these neuromodulatory mechanisms compromised by SAE jeopardizes systemic immune responses, including those of neutrophils, macrophages/monocytes, dendritic cells, and T lymphocytes, which ultimately results in a vicious cycle between brain injury and a progressively aberrant immune response. Deep insight into the crosstalk between SAE and peripheral immunity is of great importance in extending the knowledge of the pathogenesis and development of sepsis-induced immunosuppression, as well as in exploring its effective remedies.

## Background

Sepsis is one of the major threats to the survival and prognosis of patients in intensive care units (ICUs) but lacks specific and effective treatments. According to the new definition of sepsis 3.0, life-threatening organ dysfunction caused by an aberrant immune response to infection is responsible for the pathogenesis and progression of sepsis [1]. The interplay between malfunctions in the immune system and multiple organ damage is deemed the major cause of poor outcomes among sepsis cases. Maddux and colleagues reported a close association between disturbed innate immune responses and organ failure in adults with sepsis [2]. Dysfunction of multiple organs results in extensive aberration of the immune response, which may accelerate the progression of sepsis. For example, brain injury is commonly complicated by the septic state and is usually identified as the first organ exposed to an inflammatory episode [3]. Of note, sepsis-associated encephalopathy (SAE) is one of the most common etiological factors for febrile encephalopathy, especially in elderly people. Approximately 70% of patients with bacteremia develop neurological symptoms ranging from lethargy to coma, and over 80% suffer from abnormalities as measured by electroencephalogram (EEG) [3, 4]. Moreover, it has been identified that SAE is critically involved in increased mortality, extensive in-hospital cost, and prolonged hospitalization, followed by persistent cognitive impairment and limitations in physical function [5, 6]. Therefore, early recognition and prompt interference for brain injury are of great importance for the survival and prognosis of septic patients.

As we know, dysfunction of the central nervous system is responsible for the collapse of the peripheral immune system because of its central role in multiple types of neuroendocrine immune networks, including the hypothalamic-pituitary-adrenal (HPA) axis and the sympathetic and parasympathetic nervous systems. The inflammatory signals reach the different brain regions mainly through two disparate ways: the humoral and neural pathways, which involve a compromised blood–brain barrier (BBB) and activation of afferent fibers of the vagus nerve, respectively [7]. The brain further processes and manipulates the peripheral inflammatory response by initiating the neural reflex and promoting the release of neurotransmitters. For example, the cholinergic anti-inflammatory pathway (CAP), which is composed of brain cholinergic nuclei, efferent vagus nerve, and peripheral α7 nicotinic acetylcholine receptors (α7nAchRs), is reportedly beneficial for various diseases because of its anti-inflammatory capacity [8]. It has been demonstrated that activation of the CAP, either by stimulation of vagus nerve or administration of agonists for α7nAchR, significantly alleviates multiple organ damage and improves survival of septic animals [9, 10]. However, the corruption in any component of the CAP directly leads to its unresponsiveness or an aberrant response. In traumatic brain injury (TBI), for instance, the vagus nerve presents with obvious overactivity, which is responsible for the development of immune paralysis, suggesting that there is feedback for the loss of brain cholinergic nuclei [11]. The pathophysiological progression of TBI reportedly results from dysregulation of the cholinergic and inflammatory systems, while modulation of cholinergic activity shows great benefit for brain injury, and it serves as a promising remedy with neuroprotection [12, 13]. Thus, the viability and functional homeostasis of the brain cholinergic system are essential for the integrity of CAP activity. In fact, extensive apoptosis of cholinergic neurons was observed under septic exposure, which showed a close connection to an unresolved inflammatory response, as reported by Zaghloul et al., suggesting that dysfunction of the central nervous system might be an important contributor to the collapse of neuroendocrine immune networks, as well as a potential therapeutic target for sepsis-induced immune depression [14]. Even worse, brain injury might act as a vicious cycle for sepsis-induced immunosuppression because of its pivotal role in neuroendocrine immune networks.

## The role of the immune response in the pathogenesis of sepsis associated encephalopathy

**Fig. 1:**
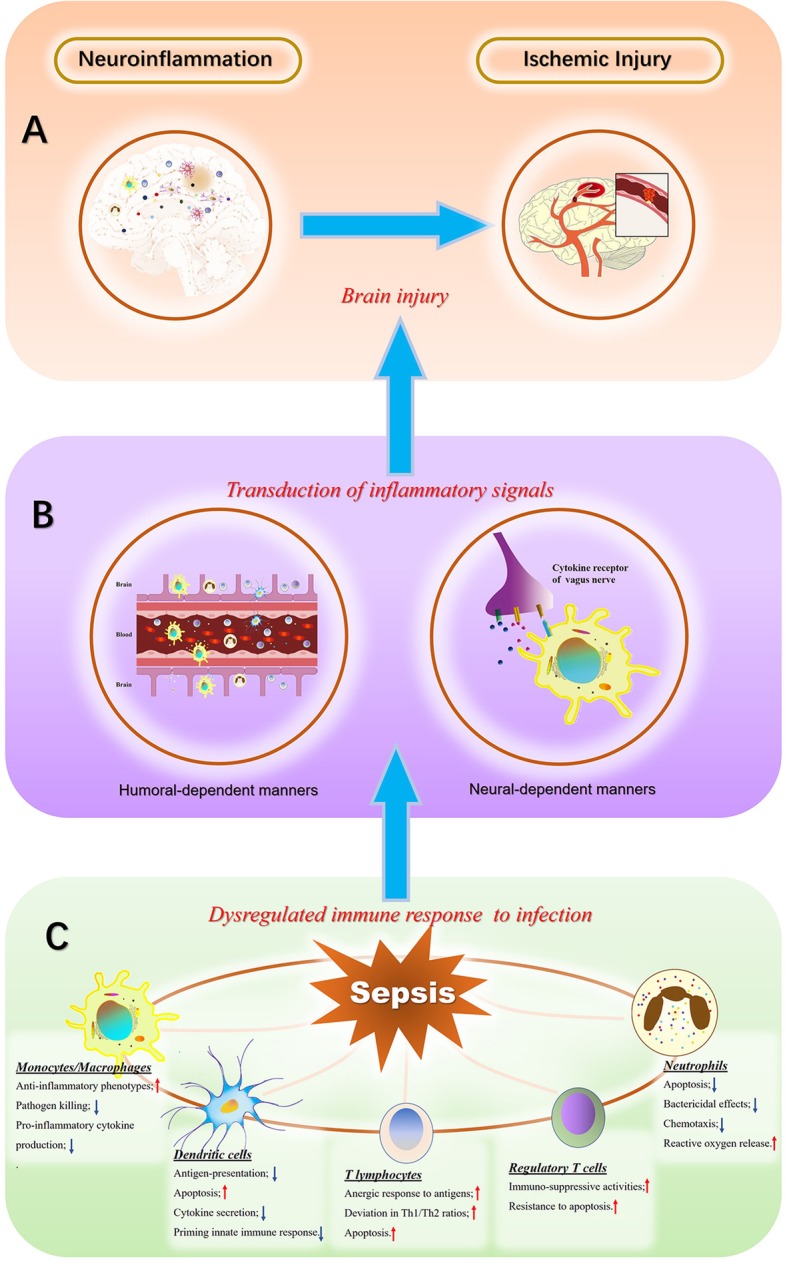
Pathogenesis of sepsis associated encephalopathy (SAE). Neuroinflammation and ischemic injury are considered as major causes of SAE (**a**), which arises from a dysregulated peripheral response to infection. The source of neuroinflammation includes both resident immune cells, such as microglia and astrocytes, and infiltration of peripheral inflammatory mediators and immune cells. In addition, inflammatory insults are responsible for the ischemic process. The inflammatory signals can reach different brain regions in both neural- and humoral-dependent manners after the initiation of septic challenge (**b**) and involve aberrant infiltration of blood–brain barrier (BBB), saturable transportation and specific areas without covering of BBB as well as neuro-inflammatory receptors. The abnormal immune response to infection is closely associated with the pathogenesis and progression of sepsis, according to sepsis 3.0 definition (**c**), and it is also a major contributor to irreversible brain damage

Multiple factors are reportedly involved in the pathogenesis of SAE (Fig. 1), including inflammatory cytokines, collapse of the BBB, ischemic processes, alterations in neurotransmitters, and mitochondrial dysfunction, while the specific mechanism has not yet been established. Two types of mechanisms have been identified with critical involvement in the development of brain injury: uncontrolled neuroinflammation and ischemic injury, which are common presentations among patients with severe sepsis [15]. Notably, the dysregulated immune response is confirmed to be a major contributor to the onset of sepsis, which highlights its pivotal role in the progression of multiple organ dysfunction syndrome (MODS), especially for the central nervous system, which is vulnerable to inflammatory insults [1, 2]. In addition, the interplay between uncontrolled inflammation and ischemic injury makes SAE a difficult issue.

### Neuroinflammatory insults

Inflammatory signals can reach different brain regions in both neural- and humoral- dependent manners after initiation of septic syndrome. For example, visceral inflammation can be detected by the vagus nerve via terminal cytokine receptors, which further stimulates brain cholinergic nuclei and acts as negative feedback for the inflammatory response by releasing acetylcholine (Ach) at the terminal efferent nerve [16]. Other mechanisms contribute to the transmission of peripheral inflammatory signals, including aberrant infiltration of the BBB, saturable transportation, and specific areas without covering of BBB [17]. In fact, the brain is the first organ that suffers from septic challenge, which might result in different impacts on the peripheral immune system in sepsis [18]. In the initial stage of sepsis, activation of the functional nucleus in the central nervous system provides an essential feedback for the peripheral inflammatory response, and it serves as a crucial interchange for multiple neuroendocrine immune networks. The CAP, as an example, has been demonstrated to have beneficial effects on various critical illnesses via inhibiting the inflammatory response [8]. However, excessive production of inflammatory cytokines, such as tumor necrosis factor (TNF)-α and high mobility group box-1 protein (HMGB1), results in deteriorative neuroinflammation and extensive loss of brain cells, as noted with ablation under specific antagonism [19, 20]. TNF-α appears to be a key mediator of SAE due to its direct correlation with BBB corruption, brain edema, neutrophil infiltration, astrocytosis, and apoptosis of brain cells, which do not occur in TNFR1-deficient mice [19]. HMGB1, commonly regarded as a later lethal mediator in sepsis, is significantly increased in different brain regions under expose to sepsis [20]. Antagonism of both blood and cerebral HMGB1 is beneficial in SAE by preventing the loss of brain cells and restoring neurocognitive function, indicating an important role for the release of inflammatory cytokines in the development of SAE [20–22].

Neuroinflammation is critically involved in the pathogenesis of SAE, as uncontrolled inflammation is a primary indicator of septic conditions. Neuroinflammation is responsible for dysfunction and massive apoptosis of brain cells, including microglial cells, neurons, and endothelial cells [23]. Both peripheral inflammation and local inflammation are induced by activation of resident brain immune cells, such as microglial cells, and astrocytes, and reportedly accounts for the induction of neuroinflammatory response and worse outcomes due to septic complications [24–26]. Over activation of microglia, for instance, is involved in the progression of brain dysfunction by deteriorating the BBB and enhancing the release of reactive oxygen species (ROS) [24, 27]. In contrast, inhibition of microglia is beneficial for abating brain oxidative damage and inflammation in sepsis, along with improvements in long-term cognitive function [24]. Astrocytes also play pivotal roles in driving inflammatory brain injury due to critical involvement in brain-immune interfaces [28]. It is reported to orchestrate the effects of immune cells in central nervous system by acting as a surveillant yet integrate center for inflammatory signals [26]. Under septic exposure, however, astrocytes present with aberrant responses that promote intractable neuroinflammation and cognitive impairment [26, 29]. The activation of astrocytes is detected in brain tissues as early as 4 h following sepsis, which attains the peak at 24 h [28]. Activated astrocytes are capable of releasing multiple kinds of inflammatory mediators, such as TNF-α, interleukin (IL)-1β, IL-6, and IL-18, and further facilitating the development of neuroinflammation, which are interpreted by enhancing expressions of p21 and nuclear factor (NF)-κB [29]. In addition, the structure of astrocytes reveals extensive changes under LPS challenge, as evidenced by structural remodeling and loss of end feet, which are responsible for the collapse of BBB [30]. Abnormal performance and loss of endothelial cells also show great potential in augmenting deterioration of the brain after the onset of sepsis through a direct link to the collapse of BBB, increased infiltration of inflammatory cells, aberrant migration of microglia, and excessive formation of ROS and nitric oxide (NO) [31]. Injury of endothelial cells as a result of neuroinflammation leads to derangement of cerebral perfusion, which renders the ischemic processes of SAE an intractable problem [32].

### Ischemic processes

Reduction in brain perfusion plays an important role in the development of SAE, and it can directly result in the abnormal cellular metabolism and oxidative stress [32]. Cerebral blood flow (CBF) is found to be significantly lower in patients with sepsis compared to that of normal controls, which shows a tight association with brain metabolism impairment [33, 34]. Similarly, cerebrovascular autoregulation presents with marked impairment and acts as one of the major triggers of SAE [35]. However, the precise mechanism of inadequate cerebral perfusion and abnormal autoregulation have not been elucidated. Multiple factors are involved in the pathogenesis of disturbed cerebral perfusion and microcirculation, such as abnormal vasoconstriction, vasopressors, decreased mean arterial pressure, and inflammatory insults [36]. The effects of other factors, including coagulation and platelets, are also noteworthy but remain controversial in the development of ischemia during SAE. A prothrombogenic phenotype is noticed at 4 h after operation by cecal ligation and puncture, accompanied by outward signs of brain dysfunction [37]. Indeed, disseminated intravascular coagulation is reportedly responsible for extensive cerebral ischemia and poor outcomes in fatal septic shock [38]. The count and mass of platelets are critically involved in maintaining the integrity of cerebral microcirculation. However, further researches did not reveal a significant difference between profound behavioral deficits and platelet recruitment in cerebral venules [37]. These conclusions were further validated by postmortem analysis of brain tissues from patients who died from septic shock. Sharshar and colleagues observed that all patients with septic shock presented with cerebral ischemia, which ranked the first in neuropathologic features, followed by hemorrhages and hypercoagulability syndrome [39]. Nevertheless, the latter two pathologies revealed no close relationship to the incidence of cerebral clotting disturbance [39]. A study by Feng et al. showed that the platelet counts were not significantly different between the SAE and non-SAE groups, or between the survival and non-survival groups in the setting of sepsis [40]. The interplay between neuroinflammation and ischemic injury is a great threat to the central nervous system following sepsis. Uncontrolled neuroinflammation jeopardizes the cerebrovascular system by inducing damage to vascular endothelial cells and imbalances in neurotransmitters, resulting in thrombogenesis and abnormal vasoconstriction, thereby further exacerbating the ischemic brain [41]. In return, the ischemic process worsens local inflammation by either increasing the infiltration of inflammatory cells or inducing dysfunction of resident immune cells that is difficult to resolve, indicating that the crosstalk between neuroinflammation and ischemic insult is a potential accelerator for the development of SAE and forms a vicious cycle [42]. Therefore, homeostasis of the immune response is of great significance in maintaining the functional integrity of the central nervous system after septic challenge.

## The impact of brain injury on the response of various immune cells

The dysregulated immune response is considered as a major cause for the development of sepsis and is a critical contributor in the pathogenesis of SAE. The influence of brain dysfunction on the systemic immune response is also a noteworthy issue for the progression of sepsis, as it plays an integral role in the neuroimmune networks (Table 1). In this regard, disruption of the HPA axis is commonly complicated by sepsis, accounts for disruption of vascular sensitivity and increases the likelihood of death [59]. The HPA axis plays an essential role in resolving systemic inflammation and immune responses by releasing glucocorticoid, while insufficient activation is considered a pivotal predictor for poor outcomes of critically ill patients [60, 61]. Therefore, reconstruction of the HPA axis may act as a promising therapeutic strategy for improving the survival and prognosis of septic patients [62]. Deep insight into the potential mechanism of the impact of brain injury on the responses of multiple types of immune cells is essential for understanding the pathophysiology of sepsis-induced immunosuppression.

**Table 1:** The function and activity of immune cells exposed to brain injury

Types of immune cells	Changes in function and activity	References
Monocytes/macrophages	Decreasing the number of monocytes; polarization of M2 phenotype; increasing production of IL-10; reducing phagocytic capability	[43], [44], [45]
Neutrophils	Impairment in ROS production; reduction of phagocytosis; shortening life-span as a result of spontaneous apoptosis	[43, 46–49]
Dendritic cells	Decreasing number of circulating DCs; anergic response to TLR3 and TLR4 stimulations; incapable of priming effective cellular immune response; disturbed infiltration and aberrant phenotypic differentiation	[50–53]
T lymphocytes	Reducing proportion of peripheral T cells; disturbing response to antigens; polarization of anti-inflammatory phenotypes; inducing imbalance between Tregs and proinflammatory phenotypes of T cells	[54–58]

### Monocytes and macrophages

The functional status and differential phases of monocytes or macrophages reveal the responsive capacity of the innate immune system, which serves as the main repository for multiple types of inflammatory mediators. Several studies have documented that monocytes and macrophages are either hyporesponsive or hyperactive during sepsis and have dysregulated cytokine production, resulting in compromised survival of septic animals [63–65]. Inflammatory monocytes and macrophages constitute a large proportion of the initial infiltrates in the central nervous system after septic exposure [66]. The activation of both infiltrated monocytes or macrophages and resident microglial cells is closely associated with excessive neuroinflammation [46, 67]. Thus, targeting the infiltration of inflammatory monocytes and inhibiting the activation of microglial cells might resolve the neuroinflammatory response and improve cognitive performance in sepsis, suggesting the potential therapeutic significance of monocytes or macrophages in SAE [46, 66].

The function and polarization of monocytes are compromised by different types of brain dysfunction. For instance, nosocomial infection is commonly complicated and regarded as a major cause of mortality secondary to severe TBI, which brings about outward signs of systemic immune depression [43, 68]. The number of monocytes was markedly decreased at 24 h post TBI, resulting in immune suppression accompanied by dominant differentiation of M2 phenotype cells [44]. Moreover, expression of intracellular IL-10 was significantly upregulated in monocytes resulting from TBI, suggesting the prominent capacity of brain dysfunction in eliciting anti-inflammatory responses [45]. Other conditions that cause a spectrum of brain dysfunction, such as neuroinflammation, brain edema, cognitive impairment, and locomotor dysregulation, involve aberrant responses of monocytes and macrophages. In addition, the phagocytic capability of alveolar macrophages was shown to be markedly reduced and augmented systemic inflammation and pulmonary damage [69]. Collectively, SAE might act as a second hit for compromised viability and the dysfunction of monocytes and macrophages in sepsis.

However, the specific mechanism underlying the unresponsiveness and apoptosis of monocytes and macrophages in acute TBI remains elusive. In physiological conditions, the central nervous system manipulates systemic inflammatory signals and immunomodulation mainly through the following three pathways: HPA axis, CAP, and sympathetic nervous system. In brain injury, however, these mechanisms act as feedback for the loss or dysfunction of central nuclei and elicit outward signs of hyperactivation, which are responsible for the abnormal response of the immune system. It has been documented that the activity of the HPA axis is enhanced after brain injury, and it relates to marked immunosuppression by increasing the production of corticosteroid [70]. A similar tendency was also observed with the CAP and the sympathetic nervous system, both of which exhibit hyperactivity during brain injury and result in immune dissonance of monocytes and macrophages. For example, the vagus nerve is overactive in brain injury, which accounts for the disturbed chemotaxis and decline in production of proinflammatory cytokines by monocytes and macrophages [11, 71]. Other factors, such as the failure in locomotor activity due to unconsciousness status, sedation, and inappropriate administration of glucocorticoids, are potential contributors to the compromised viability and function of monocytes and macrophages during SAE and should be addressed promptly.

### Neutrophils

Neutrophils are an active participant in the innate immune response and are mobilized early after infection. Reprograming of neutrophils is regarded as one of the major features of sepsis-induced immune dysfunction, as evidenced by abnormal accumulation and inefficient chemotaxis and bactericidal effects in sepsis [72]. Restoring the viability and functional homeostasis of neutrophils has beneficial effects on the survival and prognosis of septic animals [73]. Neutrophils display increased infiltration in the central nervous system and are associated with inflammatory insults at the early stage of sepsis, but they are rarely found in the normal brain [66]. Neutrophils accumulating in the central nervous system jeopardize brain cells by releasing inflammatory cytokines, increasing the activity of myeloperoxidase, and promoting oxidative damage [74]. Taken together, the balance between infiltration and withdrawal of neutrophils is of great importance for the development of SAE.

Peripheral neutrophils suffer from disorder and dysregulated apoptosis after exposure to different brain injuries. For example, in the early stage of TBI, peripheral neutrophils have remarkable infiltration in the central nervous system, which is deemed a protective behavior by eliminating damaged neurons and improving tissue repair [75]. Nevertheless, persistent accumulation of neutrophils was reported to be related to cell death, brain edema, and tissue loss, which was abated by neutrophil depletion [47]. In addition, neutrophils are implicated in early TBI and are characterized by increased ROS generation partly due to the abnormal regulatory feedback of the brain injury [48, 68]. Therefore, the vicious cycle between overactivation of neutrophils and dysfunction of the central nervous system might result in difficulties in developing efficient treatments for SAE. Persistent exposure to brain disorders, however, brings about noteworthy suppression of the function and activity of peripheral neutrophils. ROS production in neutrophils was significantly impaired in the days following brain injury, accompanied by a marked reduction in phagocytosis [49, 68]. Even though no obvious loss of neutrophil counts was found at any time point post brain injury, the lifespan of neutrophils was demonstrably shorter than those from uninjured patients as a result of spontaneous apoptosis [44, 48]. It has been demonstrated that activation of the complement system, such as C5a, is responsible for disturbed neutrophil phagocytosis that is implicated in brain injury [76], but the mechanisms of the impaired ROS production and spontaneous apoptosis remain unknown.

### Dendritic cells

Dendritic cells (DCs) are essential in maintaining homeostasis of the immune response, serving as a bridge connecting innate immunity to the adaptive immune system. Functional integrity and activity of DCs are closely related to the survival and prognosis of septic patients, while dysfunction of DCs has been identified as one of the major contributors to sepsis-induced immunosuppression and accounts for increased mortality and poor outcomes [77, 78]. DCs show evident signs of activation and maturation in the initial phase of sepsis, and this effectively primes the response of the adaptive immune system via upregulating the expression of surface molecules, including CD80, CD86, CD40, and major histocompatibility complex (MHC)-II, as well as enhancing the release of IL-12 [79]. Under persistent exposure to septic challenge, however, both the phenotypes and IL-12 secretion of DCs suffer from significant alterations, thereby resulting in disturbed antimicrobial defense [78]. The decreased number of DCs is one of the main causes of suppressed priming of the adaptive immune system, which was initially noted at 12 h post-sepsis [80, 81]. Currently, no direct evidence on the function and viability of DCs has been provided in the development of SAE, but the disorder of DCs is related to multiple organ dysfunction and poor outcomes of septic animals [82]. In addition, the abnormal response of DCs was found to contribute to the initiation and development of neuroinflammation in autoimmune encephalomyelitis, and it was also regarded as a potential therapeutic target [83, 84]. Therefore, it is reasonable to infer that dysfunction of DCs also accounts for the development of SAE due to its critical involvement in neuroinflammation.

It has been documented that the function and activity of DCs are affected by different types of brain dysfunction. In patients with severe head injury, for example, the host response to common antigens showed significant suppression, along with a marked increase in opportunistic infections [85]. The number of circulating DCs was significantly lower in patients suffering from aneurysmal subarachnoid hemorrhage compared to that of healthy controls [50]. These DCs exhibited a remarkable anergic response to Toll-like receptor (TLR)3 and TLR4 stimulation, as evidenced by diminished production of TNF-α and IL-2, suggesting that aneurysmal subarachnoid hemorrhage-induced brain dysfunction compromised the function and viability of DCs [50]. Yilmaz and colleagues found that circulating DC precursors underwent transient decrease after acute stroke, which was correlated with the severity of brain injury [51]. Furthermore, disturbed infiltration and aberrant phenotypic differentiation of DCs were also characterized under brain dysfunction [52, 53]. However, the precise mechanisms of the dysfunction of DCs and its significance in host immunosuppression remain to be clarified. Nonetheless, the inability to mount an effective cellular immune response is complicated in severe TBI, which is partly due to the impaired priming activity of DCs.

### T cells

T lymphocytes are classically representatives of the adaptive immune system, which are important for the prognosis of septic complications, and their impaired activity and aberrant differentiation are noteworthy in sepsis [86]. This indicates that the number of T cells is a prerequisite for the efficient response of the adaptive immune system. Extensive apoptosis of T cells was observed at 24 h following septic challenge, which was associated with poor outcomes and further identified as a rational and optimal therapeutic target for sepsis-induced immunosuppression, as successful remedy was achieved using anti-apoptosis drugs [87, 88]. Likewise, imbalanced differentiation of helper T (Th) cells is critically involved in the development of immune paralysis resulting from sepsis [89, 90]. Based on the types of cytokines specifically secreted by cells, Th cells are classified into three subgroups, Th1, Th2, and Th17, and proinflammatory mediators are produced by Th1 and Th17 cells, while anti-inflammatory cytokines are produced by Th2 cells. In the late stage of sepsis, Th cells are predominantly polarized to Th2 and show outward signs of anti-inflammatory status, while differentiation of Th1 cells occurs primarily during the initial phase of sepsis [91]. Failure to maintain homeostasis between Th1 and Th2 cells might be a major indicator of immune system dysfunction under septic conditions [89]. Depletion of peripheral T lymphocytes is independently associated with the development of SAE, as reported by Lu and colleagues [92]. Similarly, the types of T cells and their polarization status accounts for sepsis-induced brain injury and should be taken into consideration for effective treatments [93]. However, the specific role and significance of impaired viability and the dysfunction of T lymphocytes in the pathogenesis of SAE remain indistinct, and an imbalanced inflammatory response as a result of deviated Th1/Th2 ratios leads to brain dysfunction by augmenting local inflammation [54].

The recruitment of T cells occurs during the early stages of TBI, which controls damage and tissue repair through the release of anti-inflammatory cytokines [55]. However, the proportion and function of peripheral T cells are significantly suppressed during persistent severe head injury, followed by a marked impairment in cell-mediated immunity [56]. Previous studies showed that a reduction in peripheral T lymphocytes was observed at 24 h and remained low for 4 days post TBI, which resulted in an inadequate number of cells to exert an effective immune response [94]. Moreover, increased levels of serum catecholamines are related to a decreased percentage of peripheral T cells partly through suppressing egress from the lymph nodes due to enhanced stimulation of the β2-adrenergic receptor [95, 96]. Splenic CD4^+^ T cells play a crucial role in maintaining the integrity of the CAP, which is rendered incapable of potent anti-inflammatory effects either through splenectomy or depletion of CD4^+^ T cells [57]. Thus, a shortage of peripheral T lymphocytes due to TBI may be a vicious cycle for suppressed immune function, as it promotes feedback for the CAP by enhancing the activity of vagus nerve [11]. Peripheral blood T lymphocytes have disturbed responses to antigens and polarization of anti-inflammatory phenotypes, thereby exacerbating the undermined stability of the immune system and its ability to eliminate invading pathogens [56, 97]. Mitigation of brain injury either through downregulating neuroinflammation or reducing apoptosis of brain cells is an effective strategy for reversing the dysfunction of peripheral T cells [98].

The enhanced activity of the HPA axis and the production of glucocorticoids are theoretically responsible for the abnormal function and polarization of peripheral T cells that have anti-inflammatory capacities, which might act as feedback for the loss of effective brain nuclei [7, 99]. Regulatory T cells (Tregs), one of the major subtypes of T lymphocytes with potent anti-inflammatory activity, are extensively involved in sepsis-induced immunosuppression due to delayed apoptosis and enhanced activity [58]. Although the role of Tregs in the pathogenesis and progression of SAE has not been established, Tregs do show benefits for TBI patients via alleviating local inflammation and promoting tissue repair [100]. Nevertheless, evidence on changes in the immune function of Tregs in brain injury has not yet been provided, and deep insight into the pathophysiology of immunosuppression due to dysfunction of the central nervous system is important.

### Regulatory mechanisms

The aberrant responses of multiple neuroendocrine immune networks are reportedly responsible for the disorders in host immunity that are secondary to brain injury. The central nervous system plays an essential role in maintaining the functional integrity of the immune system, as the command center of various neuromodulatory pathways and has close connections to peripheral organs. Thus, clarifying the specific mechanism with regard to both neurologically dependent and independent pathways is of great importance for timely recognition and prompt interference in the vicious cycle between SAE and intractable immunosuppression.

#### Cholinergic anti-inflammatory pathway

CAP represents an important branch of the parasympathetic nervous system and serves as an effective therapeutic target for various inflammatory diseases. It mainly constitutes the following three parts: brain for signal integration, processing and transition, efferent vagus nerve for signal transmission, and cellular α7nAchR for initiating intracellular machinery [8]. Peripheral inflammatory signals are captured by receptors of immune-transmitter in afferent vagus nerve, such as receptors of IL-1β and prostaglandins, and then reach the nucleus tractus solitarius of the central nervous system [101, 102]. The dorsal motor nucleus is activated after interconnecting with nucleus tractus solitarius, and further drives the excitation of efferent vagus nerve for Ach release [102, 103]. The anti-inflammatory effects of CAP are achieved after activation of α7nAchR, which disrupts activation of NF-κB and inflammasomes by promoting phosphorylation of the Janus kinase 2 (JAK2)-signal transducer and activator of transcription 3 (STAT3) pathway, inhibiting the TLR4-myeloid differentiation factor 88 (MyD88)-interleukin-1 receptor-associated kinase (IRAK) cascade and the release of mitochondrial DNA [104–107]. It has been reported that activation of α7nAchR is capable of blocking activation and entry of NF-κB via interfering phosphorylation of inhibitor κB (IκB) [108, 109]. Furthermore, microRNA124 is identified involving in the anti-inflammatory mechanism of CAP after increased expression by α7nAchR activation, which is evidenced by inhibiting production of TNF-α and IL-6 [110]. Hamano and colleagues found that stimulation of α7nAchR suppressed immune responses of human monocytes by downregulating the expressions of CD14, TLR4, intercellular adhesion molecule 1, and CD40 [111]. Abnormal response of the CAP jeopardizes the peripheral immune system, and contributes to intractable immunosuppression after brain injury. For example, the vagus nerve is overactivated during the course of TBI, and it is a major cause of immune paralysis [11]. Dysfunction of the CAP is also commonly complicated in severe septic condition, as evidenced by a massive loss of brain cholinergic neurons and vagal tone together with disturbed responses of Ach-α7nAchR machinery, which fails at inhibiting inflammation and protecting organs [14, 112, 113]. A close relationship has been noted between poor outcomes and indicators of CAP malfunction, such as a decrease in heart rate variability, an increase in cholinesterase activity, and downregulation of α7nAchR mRNA expression in peripheral immune cells [113–115]. Manipulation of CAP activity through stimulating brain cholinergic nuclei or vagus nerve, inhibiting cholinesterase activity and administering agonists of α7nAchR, is beneficial for organ function and survival of septic animals by reconstruction or simulation of CAP effects [9, 10, 116, 117]. In addition, the CAP is recognized as a neuroprotective mechanism that is capable of reducing both systemic and cerebral inflammation, which further attenuates brain damage after activation [118, 119]. Intriguingly, the vagus nerve is constitutively activated in septic survivors and appears to be associated with immune impairment and increased vulnerability to infections [120]. Therefore, the bidirectional changes in CAP activity deserve timely recognition and interference to eliminate potential hazards of the immune response. In addition, the response of the CAP can be achieved by affecting the sympathetic nervous system which to some extent acts as a feedback for the circuit of CAP [121, 122].

#### Sympathetic nervous system

Activation of sympathetic nervous system (SNS) represents the “fight or flight” response to external threats. It is essential for inflammatory control and modulation of the immune response by releasing norepinephrine (NE) at terminal nerves, which is a pivotal part in the inflammatory reflex. The SNS shows critical involvement in the development and response of immune cells. For example, failure of intact SNS signaling impairs the steady-state circadian rhythmicity of hematopoietic stem cells and disturbs their mobilization [123]. Basic β-adrenoreceptor (β-AR) activity is programmed to retain lymphocytes within the lymph nodes, while activation of β-AR results in a rapid decline in lymphocytes in peripheral blood and lymphatic fluid by blocking efficient emigration [96, 124]. Further studies provided evidence that activation of β-AR in T cells promotes interactions between β-AR and C-C motif chemokine receptor (CCR)7 and CCR4 [96]. Hyperactivation of SNS has also been identified as a common characteristic of brain insults, such as TBI, cerebral ischemia and stroke, and constitutes a great threat to the immune response [125–127]. It has been demonstrated that overactivation of SNS induces extensive immunosuppression by driving a negative immunomodulatory phenotype. Di Battista and colleagues reported that exaggerated SNS activation was responsible for dysregulated production of peripheral inflammatory mediators during the course of TBI, suggesting a potential target for orchestrating the inflammatory response [125]. Activation of β2-AR by NE triggers increased levels of cyclic AMP (cAMP) and activation of protein kinase A (PKA), which results in downregulation of proinflammatory cytokines by suppressing translocation of NF-κB [128]. Moreover, enhanced SNS activity is a major cause of the decreased ratio and anti-inflammatory phenotype polarization of blood T cells through upregulating expression of cellular programmed cell death 1 (PD1) and promoting emigration and activation of Tregs in stroke [126, 129]. Furthermore, these effects are established by increasing prostaglandin E_2_ (PGE_2_) levels and disrupting the stromal cell-derived factor-1 (SDF-1) axis after enhancing β-AR activation [126]. Other signaling pathways, such as the fibroblast growth factor (FGF) 21-extracellular signal-regulated kinase (ERK) 1/2-CCL11 axis, are critically involved in SNS-driven type-2 immunity [130]. In sepsis, distinct changes in SNS activity are a failure of an efficient neuroendocrine immune network and a potential remedy for the immune response [131]. However, the regulatory mechanism underlying SNS activity in sepsis-induced immunosuppression remains largely unknown, even though its immunomodulatory impact has been extensively studied. Furthermore, no evidence has been noted implicating SNS in the pathophysiology of SAE. Given its potent immune-modulatory effect, it might be assumed that dysregulated response of SNS do jeopardize brain function by disturbing peripheral immune system during septic exposure.

#### Hypothalamic-pituitary-adrenal axis

HPA axis belongs to the neurological branch of stress system that is programmed for maintaining functional homeostasis. The hypothalamus and brain stem constitute a central component of the HPA axis and induce a cascade of endocrine hormones, including corticotropin-releasing hormone (CRH), adrenocorticotropic hormone (ACTH), and glucocorticoid [132]. The level of cortisone is a basic indicator of HPA activity and the key executor of immunomodulation. It was reported that the HPA axis is initiated early after the onset of neuroinflammation and returns to baseline levels by 24 h, which serves as a negative mechanism for the inflammatory response [133]. However, the corticosteroids are present and have ongoing release, and dominant immunosuppressive effects occur with exposure to prolonged inflammation, suggesting a pivotal role of the HPA axis in inflammatory balance [134]. The anti-inflammatory and negatively immunomodulatory effects of the HPA axis have been extensively recognized and applied in multiple diseases. Deficiency in the HPA axis, as a result of impaired production of the adrenal hormone, contributes to the deterioration of septic conditions [135]. The adrenal insufficiency is commonly complicated in critically ill patients, which presents with decreased response to exogenous ACTH stimulation from 10% to 20%, but attains 60% in patients with septic shock [60]. Activation of HPA axis is capable of restricting the inflammatory response by driving a dominant anti-inflammatory phenotype, interfering with proinflammatory intracellular signaling, and promoting negative immunomodulation [132]. For instance, stimulation of HPA system significantly downregulates NF-κB activation and further inhibits the production of proinflammatory cytokines, such as TNF-α, IL-6 and IL-1, but enhances the expression of IL-10, followed by Th2 polarization [70, 136].

Glucocorticoids reportedly enable the anti-inflammatory effects of the HPA axis by either directly forming complex with transcription factors, e.g., NF-κB, to interfere its binding with proinflammatory genes, or promoting expression of anti-inflammatory proteins, such as annexin A1, mitogen-activated protein kinase phosphatase-1 (MKP-1), and glucocorticoid-induced leucine zipper protein (GILZ) [137]. Reduction of mitochondrial ROS is also involved in the anti-inflammatory effects of the HPA axis, which is associated with upregulated expression of uncoupling protein-2 (UCP2) [138]. Over-production of corticosteroids inhibits the activity of T cells by inducing apoptosis in a Fas/FasL-dependent manner [139]. It has been documented that the HPA axis disturbs rolling and adhesion of leukocytes by reducing l-selectin activity [140]. The distribution and status of glucocorticoid receptors (GRs) also constitute important factors for the function and fitness of immune cells. Activation of GRs in DCs, for example, represses DC maturation by controlling c-jun amino-terminal kinase (JNK) activation after TLR7 and TLR8 stimulation [141]. Aberrant expression of GRs is responsible for disturbed cellular response to corticosteroids [142]. The corticosteroid system shows dysregulated performance in severe septic conditions, and it is responsible for intractable immunosuppression and poor outcomes associated with the immune response [143]. Though multiple factors are identified interfering with efficient immunomodulation of the HPA axis, the specific mechanism for dysfunction of the HPA axis under severe sepsis exposure has not been established. Indeed, abnormal response of the immune system is a great threat to the functional integrity of the HPA axis, from collapsed cerebral nuclei to dysfunction of peripheral adrenal gland during septic course [135]. It has been documented that increased production of proinflammatory cytokines, such as TNF-α and IL-1β, contributes to remarkable suppression in the production of pituitary hormones and response of corticotrope cells [144]. In addition, increased infiltration of immune cells in the adrenal gland, especially neutrophils, causes unresponsiveness of adrenal cells by inducing hemorrhages and cell death [135]. Therefore, the interplay between abnormal immune response and dysfunction of the HPA axis is a vicious cycle for collapsed modulation of the HPA axis and intractable immunosuppression secondary to sepsis. However, no direct evidence has been provided for the relationship between a malfunctioning HPA axis and SAE-associated immunosuppression. The HPA axis is noted with significant dysfunction in critically ill patients with TBI and accounts for dysregulated inflammatory responses [145]. In addition, the involvement of HPA axis in the pathophysiology of SAE should be elucidated with regard to its essential participation in modulating peripheral immune response, while it remains speculative as a result of lacking direct evidence.

## SAE is a vicious cycle for immunosuppression and a future perspective

**Fig. 2:**
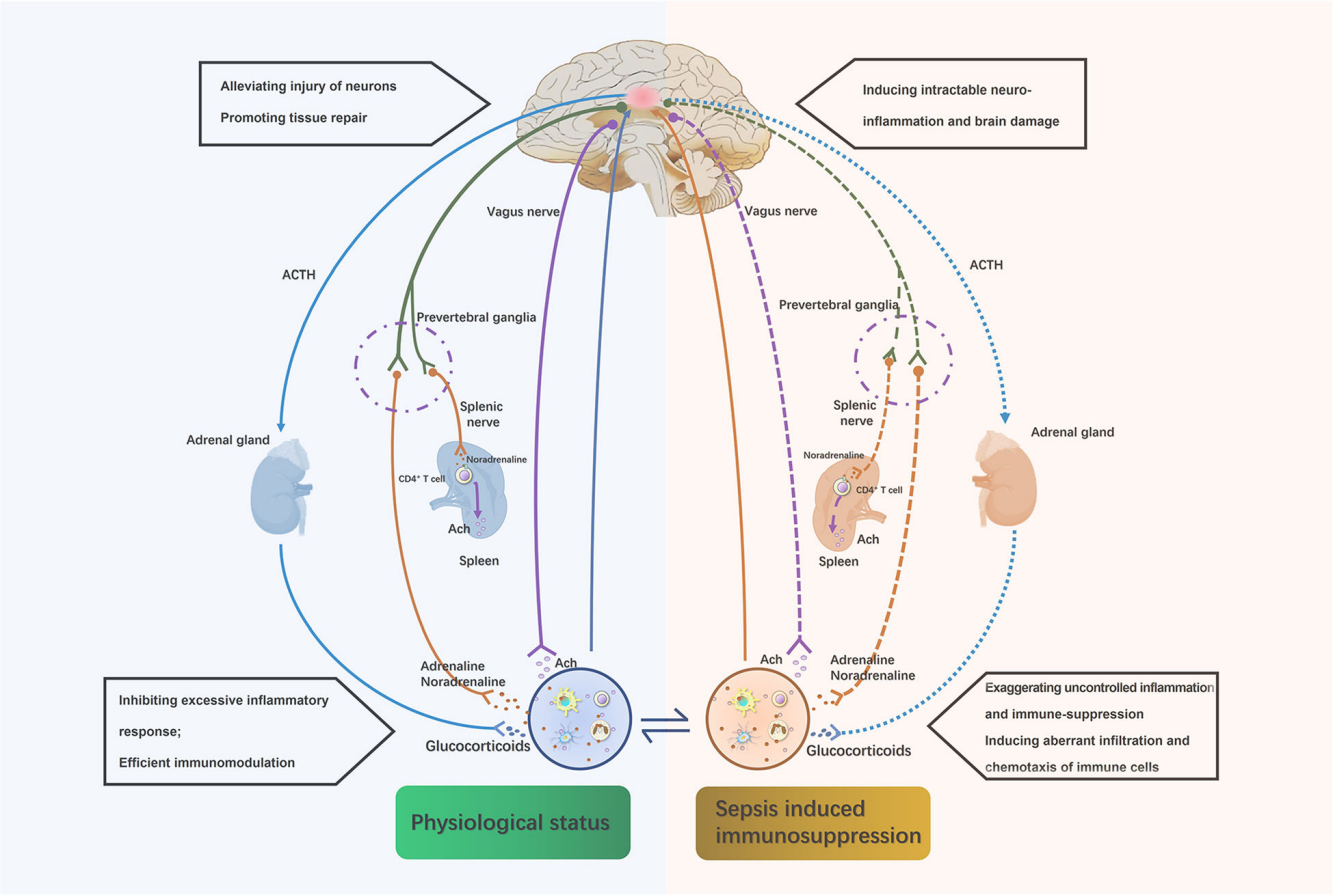
SAE acts as a vicious cycle of sepsis-induced immunosuppression. In the physiological condition, the brain is essential for the homeostasis of systemic immune response due to its central role in multiple types of neuroendocrine immune networks, including hypothalamic-pituitary-adrenal (HPA) axis, sympathetic and parasympathetic nervous system, which are capable of inhibiting excessive inflammation and enabling efficient immunomodulation. Appropriate infiltration of immune cells into the central nervous system is beneficial for functional integrity and viability of neurons. However, sepsis-induced immunosuppression contributes to intractable neuroinflammation which results in massive loss of effective nuclei and serious cognitive impairment. Furthermore, persistent brain injury impairs peripheral immune response due to abnormal response of these neuromodulatory mechanisms which present with either significant suppression or unexpected hyperactivity

**Fig. 3:**
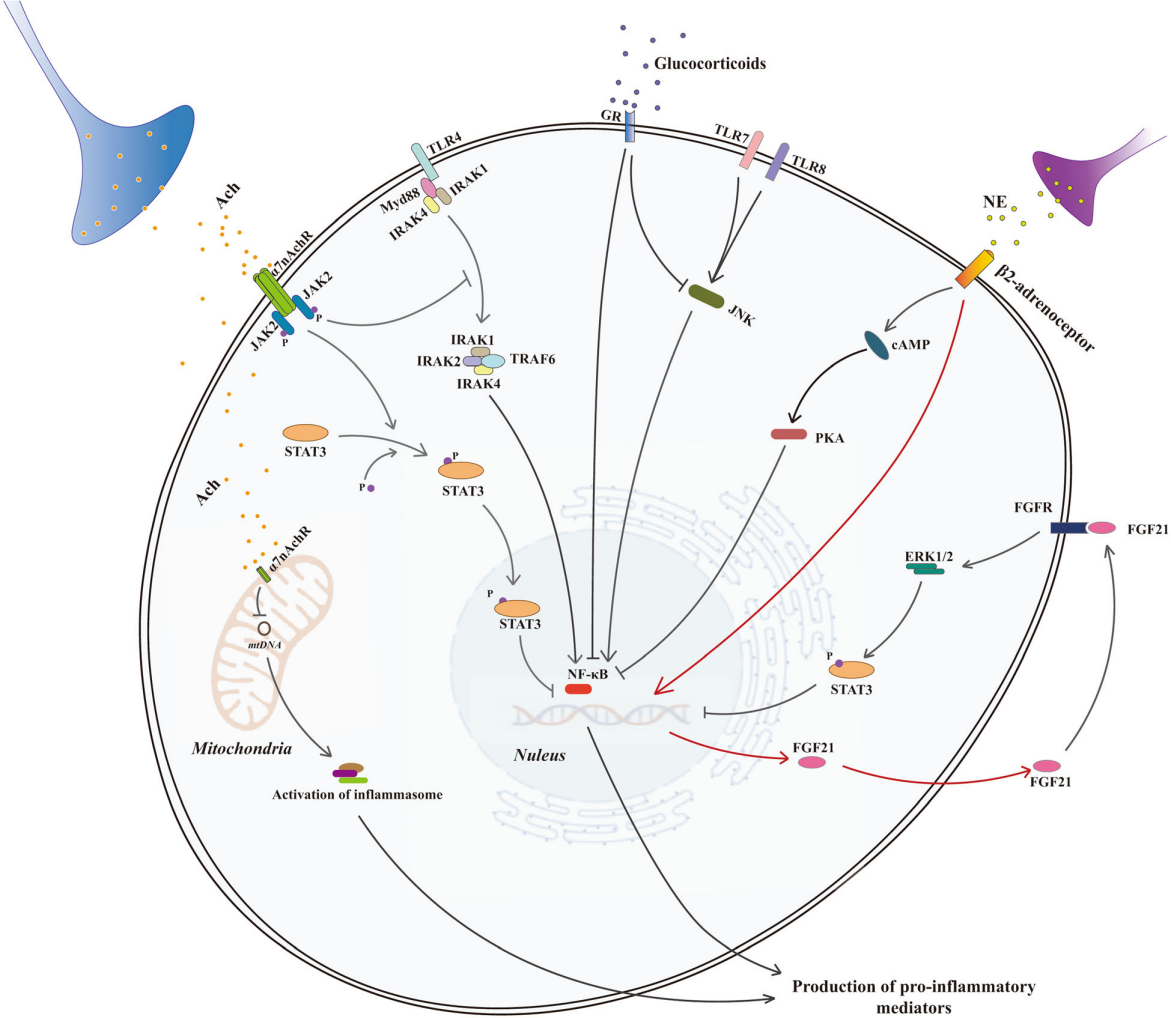
Intracellular signaling pathways of CAP, SNS, and HPA axis for anti-inflammatory responses. Acetylcholine (Ach) can be released from the terminal efferent vagus nerve and further interacts with α7 nicotinic acetylcholine receptor (α7nAChR) on immune cells. Activation of α7nAChR triggers multiple intracellular signaling pathways, including promoting phosphorylation of Janus kinase 2 (JAK2)-signal transducer and activator of transcription 3 (STAT3) pathway, inhibiting Toll-like receptor (TLR)4-myeloid differentiation factor 88 (MyD88)-interleukin-1 receptor-associated kinase (IRAK) cascade as well as declining release of mitochondrial DNA, which contribute to suppression of proinflammatory phenotypes by disturbing activation of nuclear factor-κB (NF-κB) and inflammasomes. The interaction between glucocorticoids and glucocorticoid receptors (GRs) is capable of inhibiting production of proinflammatory cytokines by downregulating NF-κB activity and disturbing c-jun amino-terminal kinase (JNK) cascade after Toll-like receptor (TLR)7 and TLR8 stimulation. Activation of β2-adrenergic receptor with norepinephrine promotes the expression of cyclic AMP (cAMP) and activation of protein kinase A (PKA), which results in decreased production of proinflammatory cytokines by suppressing translocation of NF-κB. It also increases expression of fibroblast growth factor 21 (FGF21) which further suppresses NF-κB activation by promoting activation of extracellular signal regulated kinase (ERK) 1/2-STAT3 cascades in an autocrine manner

The interplay between host immune depression and development of SAE is responsible for the deteriorating outcome of sepsis due to uncontrolled neuroinflammation and disorders of systemic immunity (Fig. 2). The infiltration of immune cells at the initial stage of sepsis is a protective mechanism for the central nervous system by eliminating damaged brain cells, maintaining homeostasis of local inflammatory response, and promoting tissue repair [23, 25, 100, 146]. Neuroendocrine immune networks are extensively activated to limit excessive inflammation and maintain balance of the host immune response (Fig. 3). For instance, stimulation of HPA axis is capable of restricting production of proinflammatory mediators and downregulating function of immune cells with proinflammatory phenotypes [137]. Persistent exposure to sepsis, however, drives irreversible brain injury by manipulating uncontrolled neuroinflammation and disturbing brain perfusion, which in turn acts as a vicious cycle for immunosuppression, as brain is the common center for multiple types of neuroendocrine immune networks [15, 18]. Sepsis-induced brain dysfunction reportedly comes with abnormal responses of these neuromodulatory mechanisms, as evidenced by either significant suppression or unexpected hyperactivity as a result of robust feedback [11, 14, 147]. For example, the HPA axis has markedly suppressed activation under prolonged exposure to severe sepsis, followed by strikingly low levels of plasma corticosterone [147]. In addition, the CAP is remarkably unresponsive due to the loss of brain cholinergic nuclei during sepsis [14]. The failure of efficient neuromodulation can lead to uncontrolled inflammation and irreversible damage to the central nervous system. Reconstruction of either HPA axis or CAP through administration of corticosteroids or stimulation of vagus efferent nerve, respectively, show great benefits for septic animals by ameliorating multiple organ damage and improving the survival [62, 120]. In fact, deteriorative neuroinflammation is the center of the vicious cycle of SAE and immunosuppression because it arises from the aberrant response of immune system and acts as a key contributor to brain dysfunction. Our previous study revealed that inhibition of cerebral HMGB1, a pivotal etiology for the late peak of inflammatory response and late mortality of septic cases, showed potent protective effects against brain injury and suppressive responses of splenic T cells [20, 148]. Taken together, these findings might provide a novel strategy not only for interfering with sepsis-induced brain injury but also a potential target for reversing intractable immunosuppression via blocking this unexpected vicious cycle.

Nonetheless, specific mechanisms of both SAE and its impacts on peripheral immune response have not yet been established, even though dysfunction of the brain has been identified, which impairs immune homeostasis and worsens outcomes in septic cases. Moreover, the specific point at which the initiation of SAE becomes a vicious cycle of immunosuppression must be clarified for timely recognition and prompt treatment. Therefore, the interplay between the pathogenesis of SAE and the abnormal response of immune cells is noteworthy and can be used for constructing a predictive algorithm after confluence analysis, given the participation of multiple immune effectors. In addition, exploration of a set would be beneficial for preventing such a vicious cycle.

## Conclusions

SAE is commonly yet severely complicated by sepsis but is prone to be neglected in clinical practice. Brain damage plays a critical role in the survival and prognosis of septic patients, which should be recognized as not only a compromised organ but also an essential participant in impaired immunomodulation secondary to sepsis because brain is the command center for multiple types of neuroendocrine immune networks, such as CAP, HPA axis, and sympathetic nervous system. The impaired activity of these neuromodulatory mechanisms reportedly contributes to decreased counts and abnormal responses of peripheral immune cells, including neutrophils, macrophages/monocytes, DCs, and T lymphocytes, which might drive brain injury into a vicious cycle of sepsis-induced immunosuppression as a result of uncontrolled neuroinflammation in the wake of progressively aberrant immunity. Therefore, it may be an efficient strategy for the sepsis-induced immunosuppressive state to block the vicious cycle between SAE and peripheral immune dissonance.

## Data Availability

Not applicable.

## Abbreviations

Ach: Acetylcholine
ACTH: Adrenocorticotropic hormone
BBB: Blood–brain barrier
cAMP: Cyclic AMP
CAP: Cholinergic anti-inflammatory pathway
CBF: Cerebral blood flow
CCR: C-C motif chemokine receptor
CRH: Corticotropin-releasing hormone
CXCR: C-X-C motif chemokine receptor
DC: Dendritic cell
EEG: Electroencephalogram
ERK: Extracellular signal regulated kinase
FGF21: Fibroblast growth factor 21
GR: Glucocorticoid receptor
HMGB1: High mobility group box-1 protein
HPA: Hypothalamic-pituitary-adrenal
ICU: Intensive care units
IL: Interleukin
IRAK: Interleukin-1 receptor-associated kinase
JAK2: Janus kinase 2
JNK: c-Jun amino-terminal kinase
MODS: Multiple organ dysfunction syndrome
MyD88: Myeloid differentiation factor 88
NE: Norepinephrine
NF-κB: Nuclear factor-κB
NO: Nitric oxide
PD1: Programmed cell death 1
PGE_2_: Prostaglandin E_2_
PKA: Protein kinase A
ROS: Reactive oxygen species
SAE: Sepsis associated encephalopathy
SDF-1: Stromal cell-derived factor-1
SNS: Sympathetic nervous system
STAT3: Signal transducer and activator of transcription 3
TBI: Traumatic brain injury
Th: Helper T cell
TLR: Toll-like receptor
TNF: Tumor necrosis factor
Treg: Regulatory T cell
UCP2: Uncoupling protein-2
α7nAchR: α7 Nicotinic acetylcholine receptor
β-AR: β-Adrenoreceptor
